# Adaptive Control of Ion Yield in Femtosecond Laser Post-ionization for Secondary Ion Mass Spectrometry

**DOI:** 10.1038/s41598-017-06562-9

**Published:** 2017-07-20

**Authors:** Dusan Lorenc, Monika Jerigova, Monika Stupavska, Dusan Velic

**Affiliations:** 1 International Laser Centre Ilkovicova 3, 84104 Bratislava, Slovak Republic; 2 Comenius University Mlynska dolina, 84215 Bratislava, Slovak Republic

## Abstract

Secondary ion mass spectrometry is an excellent technique of analytical chemistry, where primary ions sputter a solid sample generating the secondary ions which are determined. Although the ion yield is inherently low, it can be enhanced by using a post-ionization of sputtered neutral species. Our novel approach integrates this technique with a near infrared femtosecond laser post-ionization based on an adaptive control through a laser pulse shaper. The shaping of the laser pulse provides adaptive control to select a mass peak of interest and to enhance this peak intensity. Versatility is confirmed by optimizing the ion yield for different molecules (tryptophan, anthracene, polyethylene, and oxalic acid) with focus on parent ion enhancement, fragmentation process, sublimation effect, and excited secondary species. This proof-of-concept experiment provides not only a nonspecific increase of the overall ion yield, but also the selection of specific secondary species and the adaptive enhancement of their intensities on the order of 100, potentially simplifying data interpretation. Such tailored spectra might advance the (secondary ion) mass spectrometry to new capabilities.

## Introduction

**Figure 1:**
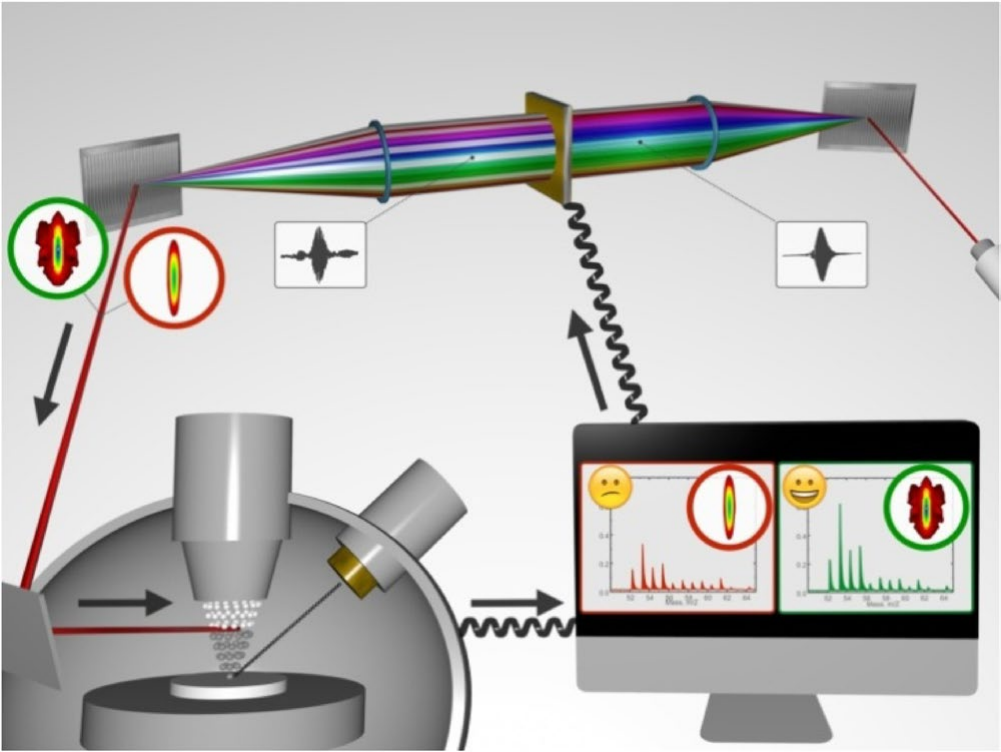
SIMS (lower part, left side), with primary ions, sputters the secondary species, which are post-ionized with the laser pulse and then detected. The laser pulse (upper part, right side) is generated and then shaped with the pulse shaper (upper part, middle). The evolutionary algorithm optimizes the mass spectrum (lower part, right side) by changing the pulse phase. The shape is represented with the FROG trace (unshaped and shaped in red and green circles, respectively). The closed loop adaptive control (arrows) consists of the SIMS, the shaper, and the algorithm. Note that Dr. Daniel Repovsky, the copyright holder of the image, granted a permission to publish.

Secondary ion mass spectrometry (SIMS)^1–4^, using primary ions to sputter secondary ions, provides a highly sensitive and comprehensive chemical spatial analysis of almost all solid samples. Different samples result in different secondary ion yields due to the matrix effect^4^, which limits quantification. Moreover, the secondary ions represent only few percents of the sputtered species, while the majority is neutral, forming a base for secondary neutral mass spectrometry (SNMS). While in SIMS the secondary ions originate from the sputtering/ionization processes at the surface, SNMS uses an external source, usually a laser, to post-ionize the sputtered neutral species^5–20^. The laser post-ionization utilizes either resonant or non-resonant mechanisms^4^, with pulses ranging from nanoseconds to femtoseconds (fs), and with visible, ultraviolet or near-infrared (NIR) wavelengths. The NIR fs laser provides an attractive alternative to non-resonant/nonspecific post-ionization by increasing the molecular parent ion yield, as well as suppressing molecular fragmentation^21^. The nonspecific post-ionization is based on strong field effects such as multiphoton ionization (MPI) and tunnel ionization (TI), where the processes of multiphoton absorption and potential energy surface bending are operative, respectively. Our novel approach is advancing the SIMS/SNMS post-ionization by combining the broadband fs laser with an adaptive control through a laser pulse shaper. The adaptive control combines the shaper with an evolutionary algorithm. The shaper, as a spatial light modulator, provides masks of liquid crystal pixels, which shape the laser phase and/or intensity. The shape of laser pulse is usually characterized by a frequency resolved optical gating (FROG) trace^22^. The intensity of mass peak is a parameter for a fitness function of the evolutionary algorithm. The algorithm optimizes the adaptive control and changes the masks in order to maximize the mass peak intensity^23^. Although, objections against coherent control were suggested^24^, our adaptive control was based on MPI or TI and does not necessarily claim a coherent control. This combination represents a novel concept in the SIMS/SNMS technique, providing not only an overall increase in the secondary ion yield, but also a selective enhancement of the specific molecular ion yield, giving us the best of both worlds - the nonspecific increase and the adaptive enhancement. Our proof-of-concept experiment successfully integrated the SIMS with the NIR fs laser, the laser pulse shaper, and the evolutionary algorithm^25^, as illustrated in Fig. 1.

This method might not only reduce the complexity of mass spectra, but also uses only one laser. Several molecules (tryptophan, anthracene, polyethylene, and oxalic acid) were chosen for these proof-of-concept experiments, in order to elaborate on the enhancement of a molecular parent ion yield, a suppression of molecular fragmentation, a sublimation effect of the samples, and the secondary species which might be excited.

## Results and Discussion

The primary ions, colliding with tens of thousands eV, deposit a great amount of energy into the sample. However, this energy, besides that which is used for ionization and sputtering^26–29^, is still difficult to study^28, 29^. The secondary species can be vibrationally and/or electronically^29^ excited, which might not influence the SIMS spectra, but can affect the post-ionization. The vibration excitation^30^ can increase a phonon-induced (thermal) desorption/sublimation during SIMS. The electron excitation can be crucial since the adaptive control post-ionization, through the multiphoton or tunnel ionizations, is dependent on the actual potential energy surface.

**Figure 2:**
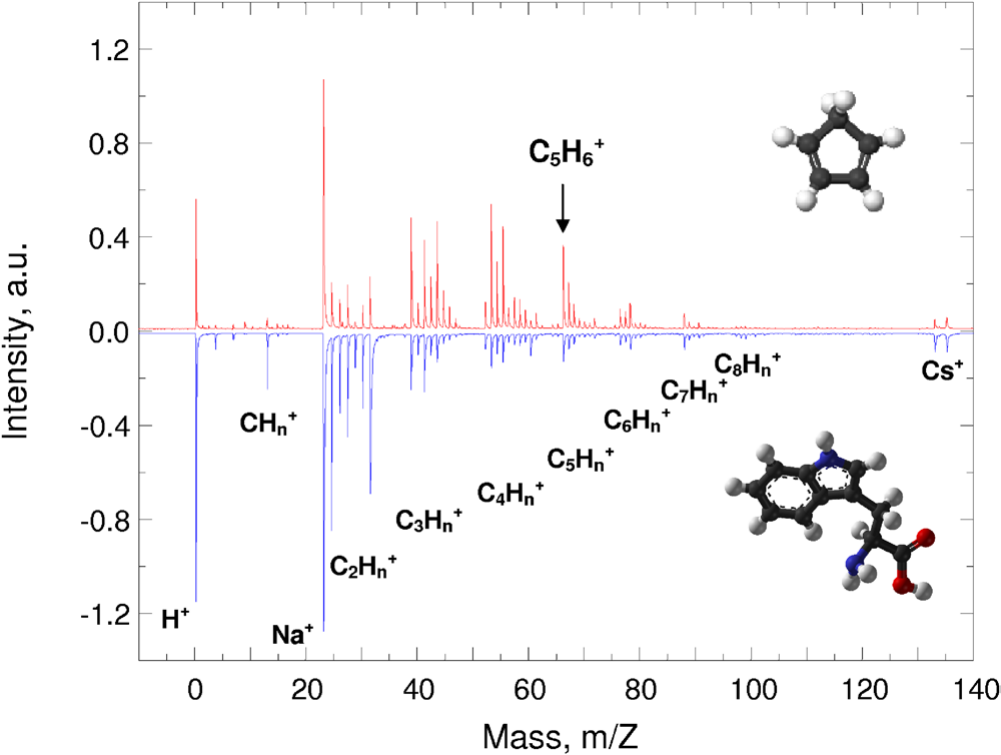
Tryptophan post-ionized mass spectra with unshaped pulse (a lower part in blue) and adaptively controlled post-ionized mass spectrum, with focus on the C_5_H_6_ fragment, with optimized shaped pulse (an upper part in red). Note that the spectra are mirror imaged for clarity to compare the intensities. The molecular structures of tryptophan and C_5_H_6_ fragment as insets in the lower and upper parts, respectively.

The adaptive control was initially tested on tryptophan (C_11_H_12_N_2_O_2_, mass 204 a.u.), which is a relatively complex molecule with both aromatic/cyclic and linear parts, which demonstrated different roles in the adaptive control^31^. Although tryptophan was cooled down to 150 K, in order to minimize its sublimation, the minor part of the post-ionization signal might have originated from the gas phase. The post-ionized mass spectrum with the unshaped laser pulse and the adaptively post-ionized mass spectrum with the optimal shaped laser pulse are shown in Fig. 2. Importantly, the original SIMS signal was maintained at a minimal level (the noise level) in order to observe exclusively the post-ionization processes. The post-ionized spectra in Fig. 2 provided H^+^, Na^+^, and the groups CH_n_^+^, C_2_H_n_^+^, C_3_H_n_^+^, C_4_H_n_^+^, C_5_H_n_^+^, C_6_H_n_^+^, C_7_H_n_^+^, and C_8_H_n_^+^. These groups of ions, consisting of C, H, O and N, were assigned as only C_x_H_n_^+^ ions. This simplification was used only for figure clarity; every studied mass peak was determined exactly.

The molecular fragment ion C_5_H_6_^+^ at m/z 66 was focused on and the result of the adaptive control is shown in Fig. 2, the upper part, as well as its tentative structure as the inset. Note that other results are summarized in Table 1 and Supplementary Information. The C_5_H_6_^+^ fragment was purposely selected for the following reasons. Firstly, although it is not a molecular parent ion, it is a relatively large molecular fragment ion. Secondly, the initial intensity was relatively low, to best test the enhancement effect. Thirdly, the ion is in the middle of significant peaks C-C_8_, representing the average mass. Fourthly, the molecular structure of C_5_H_6_^+^ can be assigned as a cyclic form with double bonds. Fifthly, such a cyclic structure is more stable for the adaptive control than the linear part which readily fragmented^31^. Sixthly, such a molecular structure can also be assigned as a neutral independent molecule; however, it can originate from the neutral fragmentation of tryptophan and only then is post-ionized. Seventhly, such a molecular structure can represent a molecular fragment ion originating from the initially post-ionized, and then fragmented, tryptophan molecule. The lower part of Fig. 2 shows that the initial post-ionization of the tryptophan species resulted in an exponential-like decreasing ion intensity dependence from H^+^ to C_8_H_n_^+^, similar to SIMS, generating small fragments, with a higher probability, than it maintains the intact molecule. Interestingly, the adaptive optimization changed this dependence, as demonstrated in Fig. 2. The upper part of Fig. 2 shows the overall enhancement of C_5_H_n_^+^, which is also associated with the enhancement of C_4_H_n_^+^ and C_3_H_n_^+^. On the contrary, the C_2_H_n_^+^, CH_n_^+^, and even the H^+^, ion intensities decreased during the same process. These results suggest that the adaptive control of the larger stable fragment might suppress the molecular fragmentation. These results also support the different roles of the aromatic/cyclic and linear parts of the molecule in the adaptive control^31^. The molecular fragments of C_5_H_n_^+^, C_4_H_n_^+^, and C_3_H_n_^+^ can be assigned as cyclic stable structures of suitable robustness in the adaptive control. On the contrary, the molecular fragments of C_2_H_n_^+^, CH_n_^+^, and H^+^ can only be assigned as linear fragments. Both of these groups then play different roles in the ionization and fragmentation processes. Note that the C_4_H_4_^+^ fragment ion at m/z 52 was also studied, and by enhancing either the C_5_H_6_^+^ or C_4_H_4_^+^, the intensities of both increased, while in both cases the intensities of the C_2_H_n_^+^ and CH_n_^+^ decreased. These results suggest that the adaptive control is sensitive to the structure of molecules and/or fragments, or eventually to the original position within the intact molecule; with a potential to differentiate isomers. In any case, to differentiate between sequences of fragmentation/ionization and ionization/fragmentation is not trivial.

**Table 1:** A list of molecules and their molecular parent and molecular fragment cations with their adaptively controlled enhancement factors.

Molecules	Parent Ion (*Enhancement factor*)	Fragment Ions (*Enhancement factor*)
Tryptophan	—	C_4_H_4_ (*2*.*0*); C_5_H_6_ (*3*.*0*)
Anthracene	—	C_3_H_3_ (4.0); C_4_H_4_ (*8*.*0*)
Polyethylene	—	C_2_H_2_ (*1*.*3*); C_3_H_6_ (*2*.*7*); C_4_H_8_ (*1*.*7*)
Oxalic Acid	C_2_H_2_O_4_ (*3*.*0*)	—

**Figure 3:**
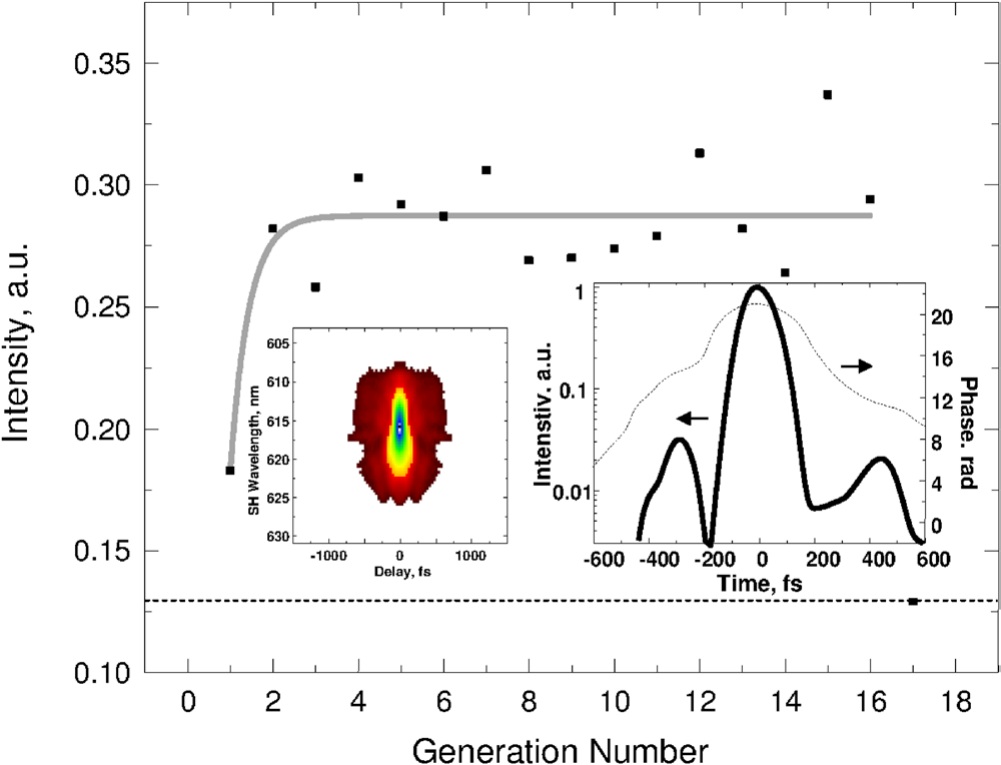
The intensity of tryptophan fragment ion C_5_H_6_ as a function of optimization generations. The fitting solid line is only to guide the eye. The insets represent the optimal FROG trace (left) and the optimal intensity and phase of the shaped pulse (right).

In the closed loop adaptive control process, the integral intensity of the C_5_H_6_^+^ peak was selected as the parameter in the evolutionary algorithm. The enhancement was related to the post-ionized intensity by using the unshaped pulse as shown in Fig. 3 as a dashed line. Note that the unshaped pulse has a centro-symmetric trivial FROG trace, as shown in Fig. 1 (in the red circles). The evolutionary algorithm was then applied to shape the laser pulse phase with the shaper in order to maximize the C_5_H_6_^+^ peak intensity. The enhancement factor of approximately 3 was reached after several generations of the adaptive control, as shown in Fig. 3. The FROG trace of the optimized shaped pulse, as shown in the inset of Fig. 3 on the left side, is clearly non-trivial. The FROG provides a color scale of the intensity as a function of the time delay and optical wavelength, determined as second harmonic (SH) generation, within the pulse. The additional dependence of phase and intensity as a function of the time delay is shown in the inset of Fig. 3 on the right side.

**Figure 4:**
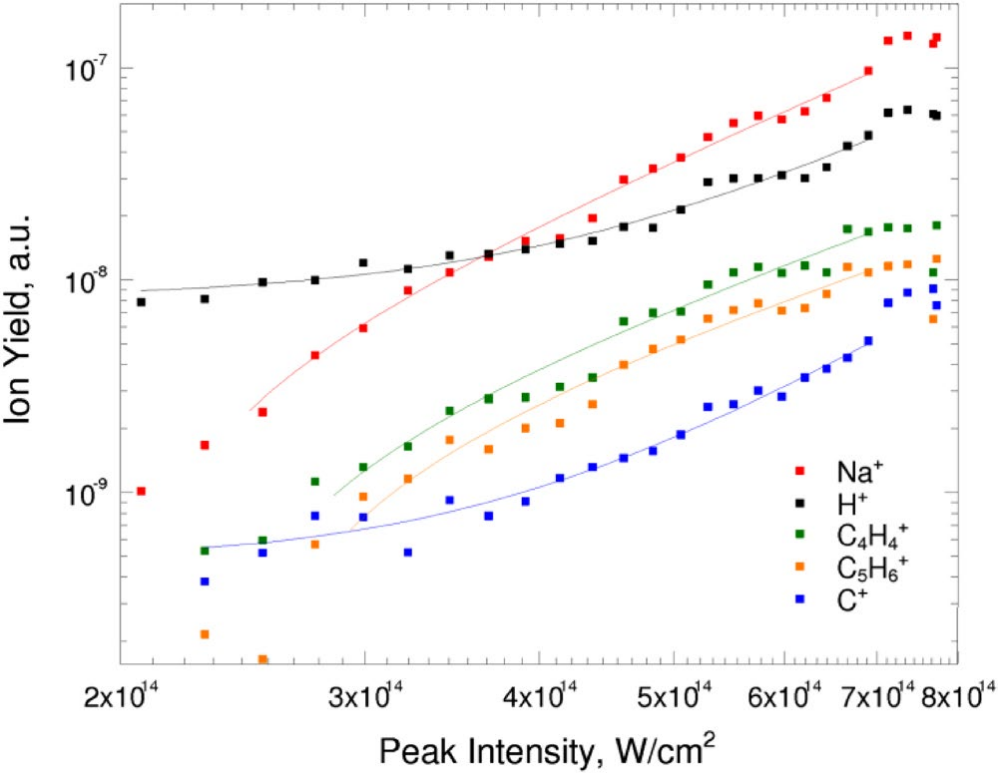
The data points of ion yield for tryptophan ion species and Na cation as a function of the unshaped laser peak intensity. The lines correspond to the fits of MPI model.

The dependences of the tryptophan C_5_H_6_^+^, C_4_H_4_^+^, C^+^, H^+^ ion yields and also the Na^+^ ion on the laser peak intensities were determined and are shown in Fig. 4. A simple reduction of laser peak intensity during the optimization generation, shifting conditions from “less” TI to “more” MPI^32^, cannot account for the observed enhancement since the C_5_H_6_^+^ yield decreases monotonically with the decreasing intensity. Moreover, the adaptive control experiments were performed with shaping the pulse phase only, maintaining the pulse intensity constant. Within the intermediate range of laser pulse intensities, i.e. from 2 × 10^14^ W/cm^2^ to 8 × 10^14^ W/cm^2^, the data points were fitted with the MPI model, as shown in Fig. 4 as lines. Note that all optimization generations were performed within this range. The general scaling law for MPI probability^33^ was applied to fit the laser intensity dependent ion yield:

1 $$P \sim \sigma {^{\prime} }_{N}{I}^{N}$$

where *σ*′_*N*_ is the *N*-photon ionization cross-section, *I* is the incident laser peak intensity, and *N* is the number of photons required for the ionization. The energy amount of 3.5 photons was required for the low mass fragment of C^+^ and 2.2 photons for the heavier fragment of C_5_H_6_^+^. The species of C_5_H_6_^+^, C_4_H_4_^+^ and C^+^, H^+^ represent groups of the larger and the smallest fragments, following the same trends of “saturating” and “increasing” dependences, as shown in Fig. 4, respectively^34^.

**Figure 5:**
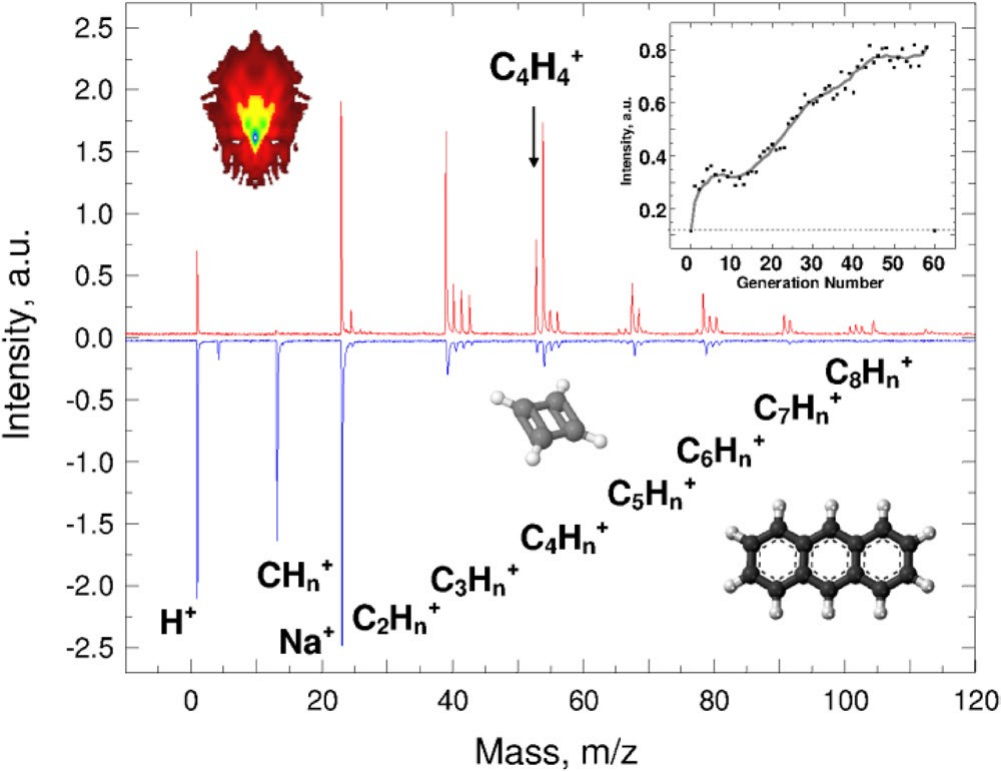
Anthracene mass spectra, post-ionized with unshaped (blue) and optimal shaped pulse (red) for the C_4_H_4_ fragment. Left and right upper insets provide the FROG trace and the enhancement factor determination, respectively. The molecular structures of anthracene and C_4_H_4_ fragment as insets in the lower right and left parts, respectively.

Since the adaptive control for SIMS was adopted from the gas phase experiments^9, 31^ anthracene (C_14_H_10_, mass 178 a.u.), as one of the volatile and extensively studied molecules, was also tested, as shown in Fig. 5. Although the sample was cooled down to 150 K, a very significant part of the post-ionization signal originated from the sublimated anthracene into the gas phase during the SIMS measurement. The molecular fragment ion of C_4_H_4_^+^ was optimized with the enhancement factor of 8.0 by using the specific and non-trivial FROG trace of the shaped laser pulse, as shown in Fig. 5 (left side inset). Note that moreover, the C_3_H_3_ fragment was also chosen to represent a potential neutral radical, which was also adaptively controlled; the spectra are provided in Supplementary Information. The C_4_H_4_^+^ fragment of anthracene was chosen in order to directly compare it with the C_4_H_4_^+^ fragment of tryptophan. Both have identical masses, possibly identical structures, but quite different enhancement factors of 8.0 and 2.0, respectively. Since their post-ionization conditions are the same, the different enhancement factors might then shed some light on the role of the extra internal energy of molecule. The differences are in the structures of the original molecules and in the processes used to transfer them into the gas phase. The tryptophan species were generated mostly from the SIMS sputtering process; they were potentially excited in the collisions of the SIMS mechanism and presumably left at the higher electron/vibration excited states. The anthracene species were generated mostly from the sublimation process and presumably left the surface with low thermal energy and at electron ground states. If these electron/vibration excited and low thermal states are assumed, a more complex process of the adaptive control might be expected. Indeed the enhancement factor of approximately 3 was reached within 5 generations and then after 50 generations, the enhancement factor increased at the value of approximately 8, as shown in the right upper inset of Fig. 5. These observations might suggest that the initial optimization was mostly performed on sputtered species, as in the case of tryptophan, with a similar enhancement factor of 2, and then the following optimization was utilized on sublimated molecules. These results indirectly suggest that the adaptive control might be sensitive to the electron/vibration^35^ excitations and it presumably performs better on the ground state species than on the excited state species due to their basic and complex potential energy surfaces, respectively.

Polyethylene (monomer C_2_H_4_, mass 28 a.u.) was chosen as a case of a sample with virtually no gas phase background from the thermal desorption/sublimation process, contrary to the case of anthracene. The adaptive control was performed on the fragment of C_2_H_2_^+^, linked together as a group with C_3_H_6_^+^ and C_4_H_8_^+^ fragments. The group optimization of similar fragments resulted in comparable enhancement factors of 1.3, 2.7 and 1.7, respectively. Oxalic acid (C_2_H_2_O_4_, mass 90 a.u.) was chosen as a case of a small compact molecule and the adaptive control was targeted to optimize the intact molecular parent ion yield. The spectra were assigned with ions of H^+^, CH_n_^+^, O^+^, Na^+^, CH_n_O^+^, C_2_H_n_O^+^, and C_2_H_n_O_2_^+^. The intensity of the molecular parent ion C_2_H_n_O_2_^+^ increased by the factor of 3.

## Conclusion

In conclusion, our proof-of-concept experiment successfully integrated the adaptive control in femtosecond laser post-ionization through the programmable laser pulse shaper into the SIMS technique. Besides the observation and determination of the enhancement factors, the stabilization of the larger fragments, the suppression of the smaller fragments, the differentiation between the sputtered and sublimated species, the dependence between the excited and ground states, the group optimization of the linked mass peaks, the optimization of the neutral radical, and finally the optimization of the intact molecular patent ion were achieved and discussed. Note that since we kept SIMS signal on the minimal level, might be 1%, we have collected post-ionized signal, as 99% of the signal. Therefore the enhamcement factors between 1.3 and 8.0, as the comparison between the ushaped and shaped laser pulses, are in reality approximately between 130 and 800 in comparison with the SIMS signal only, respectively. This method seems to have the potential to advance the SIMS/SNMS techniques.

## Methods

An experimental setup consisted of the NIR fs laser, the programmable liquid crystal pulse shaper, and the TOF(time-of-flight)-SIMS mass spectrometer within the closed loop control of the evolutionary algorithm. The chemicals tryptophan, anthracene, and oxalic acid were purchased at ≥99% purity in powder form. The samples were prepared as pellets without previous purification. The pellets were processed in hydraulic press under the pressure of 98 N.cm^−2^ for 2 minutes. Polyethylene was purchased in the form of pellets and was analyzed without further processing.

### Near-infrared femtosecond laser

The NIR fs Cr:forsterite master oscillator power amplifier with a three-stage amplifier was used as a laser source for post-ionization (110 fs, 4.5 mJ, 1240 nm, photon energy ~1 eV). The size of the output laser beam was reduced using a 2.5:1 telescope in order to match aperture of the all-reflective pulse shaper. After leaving the pulse shaper, the beam passed through a 4:1 expander and was subsequently focused by a 300 mm lens (f10) into the SIMS chamber within approximately 500 micrometers over the sample surface. A typical laser peak focal intensity was of the order of 1 × 10^14^ W/cm^2^. Real time pulse diagnostics was performed by using GRENOUILLE FROG (Swamp Optics) and QuickFrog software. Although the SIMS standard operation mode employs a repetition rate of 10 000 Hz, the experiment was carried out at 50 Hz which substantially reduced the total yield of all ions in the spectrum, limited by the laser repetition rate. Note that the temporal resolution of the experiment is currently limited mainly by the mutual temporal jitter laser-SIMS being of the order of 1 ns while SIMS has estimated intrinsic temporal jitter of the order of 0.1 ns. The laser beam spatial profile was characterized using a pair of prisms and a camera DCC1645C-HQ and obtained intensities were cross checked with the calibration obtained by comparison to the appearance thresholds for multiply charged Xe, e.g.

### Secondary ion mass spectrometry

The sample surface was bombarded with 25 keV Bi_3_^+^ primary ions (SIMS IV, IONTOF) with the pulse duration of 1000 ns and a repetition rate of 50 Hz. The mass spectra were sampled using digital oscilloscope (Wavesurfer 422, LeCroy) and subsequently downloaded for further processing, averaging 250 spectra for each scan. The obtained spectra were calibrated against the standard SIMS spectra.

### Adaptive control

The closed loop adaptive control employed a grating based all-reflective mode pulse shaper (Proteus Optics) and the loop was closed by taking the input to the TDC discriminator of SIMS. The mass spectra were displayed on the oscilloscope and the evolutionary strategy was then applied to the mass peaks of choice. A phase only shaping was applied in order to avoid the uncertainty that would otherwise arise if both intensity and phase shaping were applied. An initial set of 10 to 15 random masks was prepared with four pixels tied together to reduce a size of search space. The fitness function was calculated for each spectrum corresponding to a specific mask and the best two masks were chosen using proportional selection for propagation. New masks were generated using operators including mutation and crossover with 2.5% mutation rate. Changes resulting from the evaluation of the fitness function were subsequently applied to the liquid crystal mask of the spatial light modulator. Pixel tying was implemented in order to reduce the search space of solutions. Selected optimization generations were cross checked against the “untied” pixels option thereby providing access to the whole resolution of the pulse shaper and no significant differences were observed compared to the reduced search space case.

## Electronic supplementary material

Supplementary Information
