# Supplementary material for: Adaptive Control of Ion Yield in Femtosecond Laser Post-ionization for Secondary Ion Mass Spectrometry
Source: Sci Rep. 2017 Jul 20;7:5953. doi: 10.1038/s41598-017-06562-9 (PMC5519762; doi:10.1038/s41598-017-06562-9)
Supplement: Supplementary file 1 — Supplementary Information [file 41598_2017_6562_MOESM1_ESM.doc]

**Adaptive Control of Ion Yield in Femtosecond Laser**

**Post-ionization for Secondary Ion Mass Spectrometry**

Dusan Lorenc,^[a]^ Monika Jerigova,^[a,b]^ Monika Stupavska,^[b]^ and Dusan Velic*^[a,b]^

[a] Prof. Dr., Dusan, Velic *
Dr., Dusan, Lorenc
Dr., Monika, Jerigova
International Laser Centre
Ilkovicova 3, 84104 Bratislava, Slovak Republic
E-mail: velic@ilc.sk

[b] Prof. Dr., Dusan, Velic
Dr., Monika Jerigova
Dr., Monika, Stupavska
Comenius University
Mlynska dolina, 84215 Bratislava, Slovak Republic

[
D.L. and M.J. equally participated

plus Supplementary Information


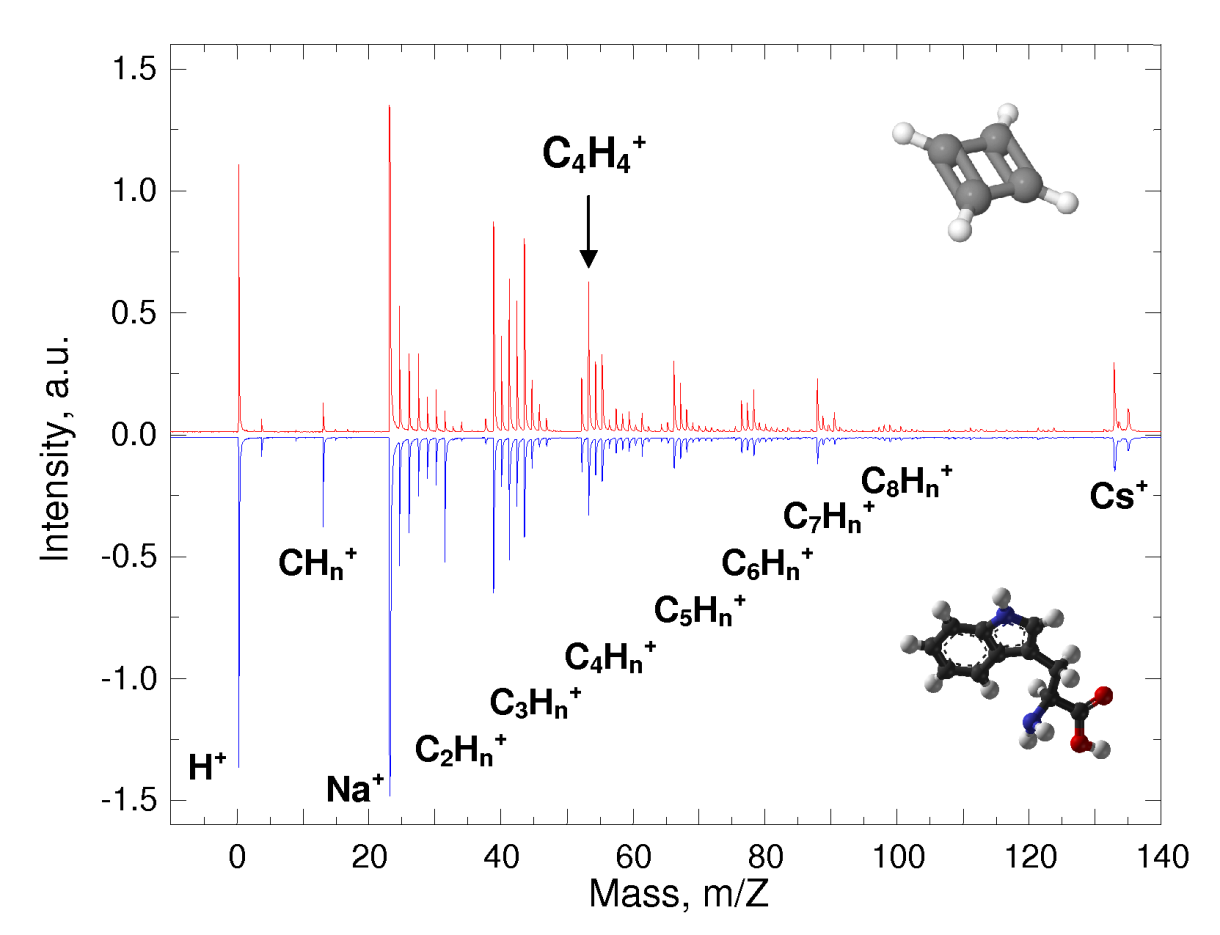


**SI.1.1** Tryptophan mass spectrum post-ionized with unshaped pulse (lower part in blue), the molecular structure as the inset, and adaptively controlled mass spectrum with focus on the C_4_H_4_ fragment with optimized shaped pulse (upper part in red), the tentative structure of the fragment as the inset.


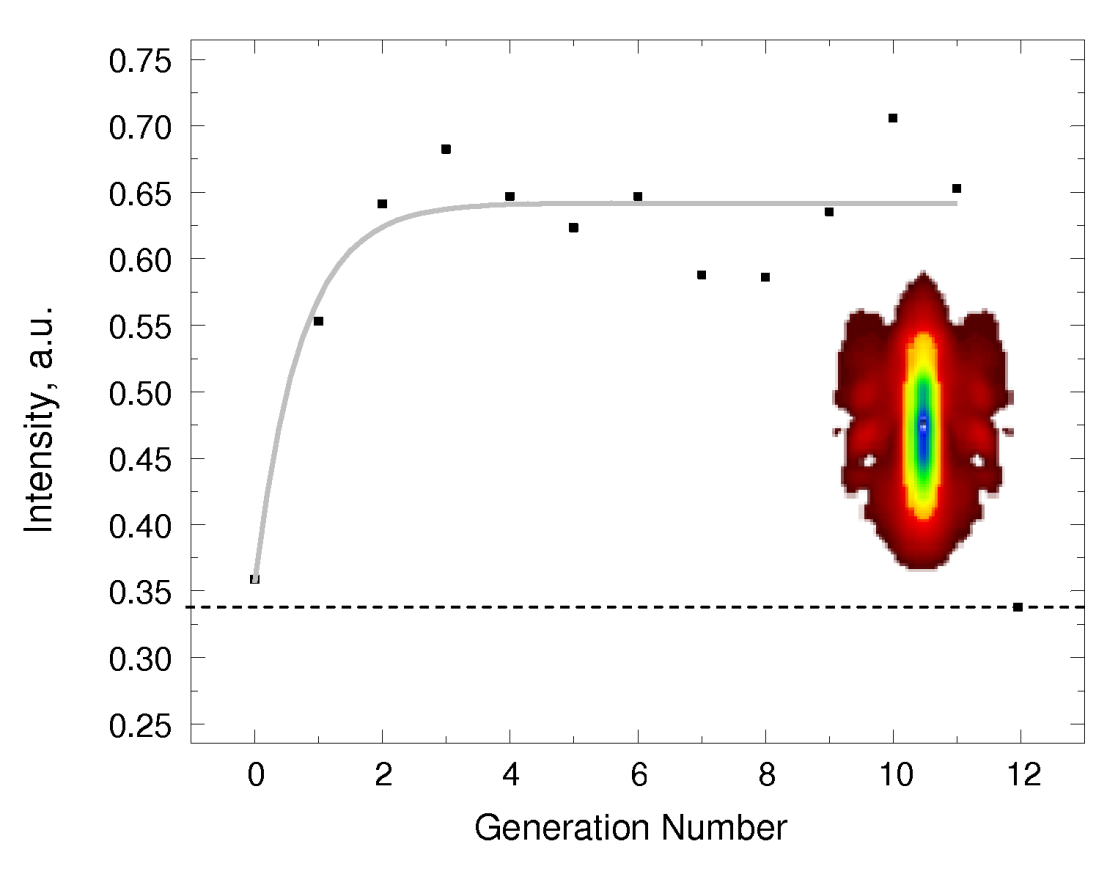


**SI.1.2** The intensity of tryptophan fragment ion C_4_H_4_ as a function of optimization generations. The fitting solid line is only to guide the eye. The enhancement is related to the post-ionized intensity by using the unshaped pulse as shown as a dashed line. The inset is the optimal FROG trace, providing a color scale of the intensity as a function of the time delay and optical frequency within the pulse.


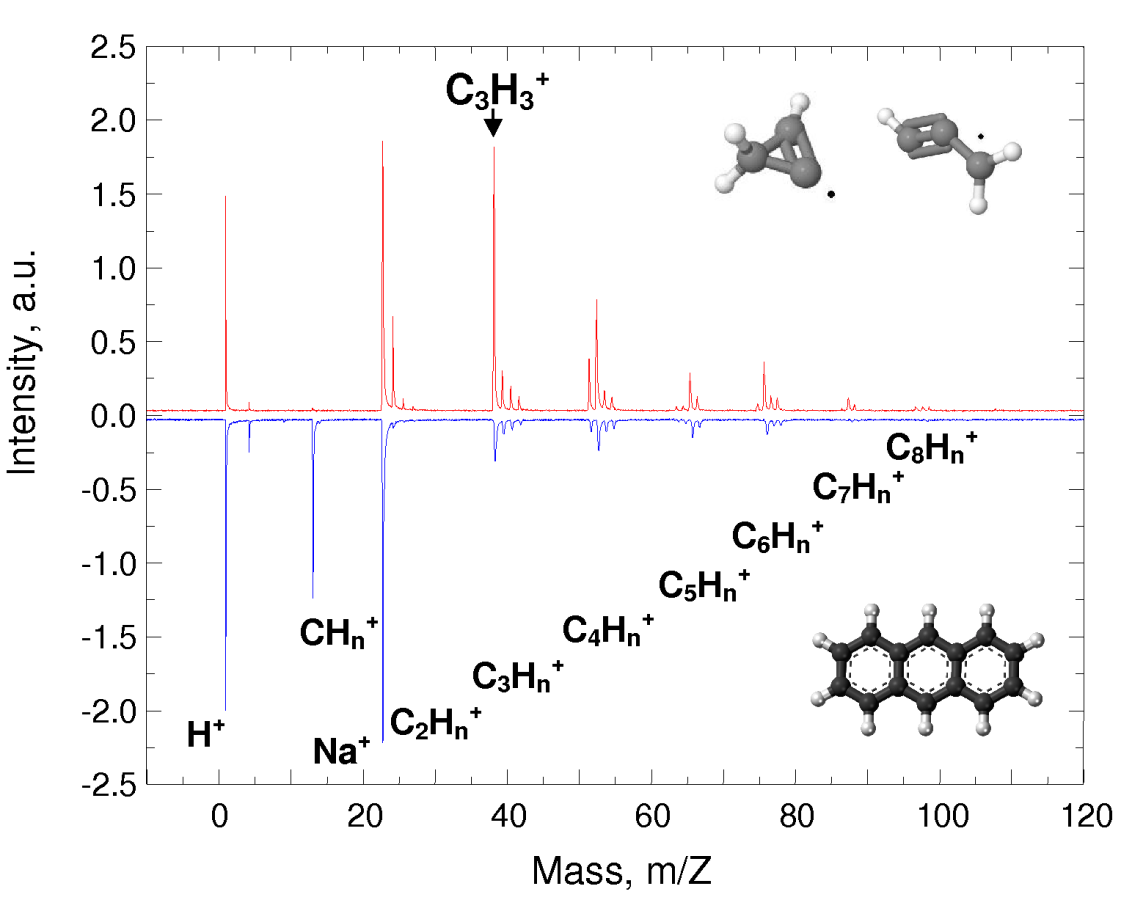


**SI.2.1** Anthracene mass spectrum post-ionized with unshaped pulse (lower part in blue), the molecular structure as the inset, and adaptively controlled mass spectrum with focus on the C_3_H_3_ fragment with optimized shaped pulse (upper part in red), the tentative structures of the fragment as the inset.


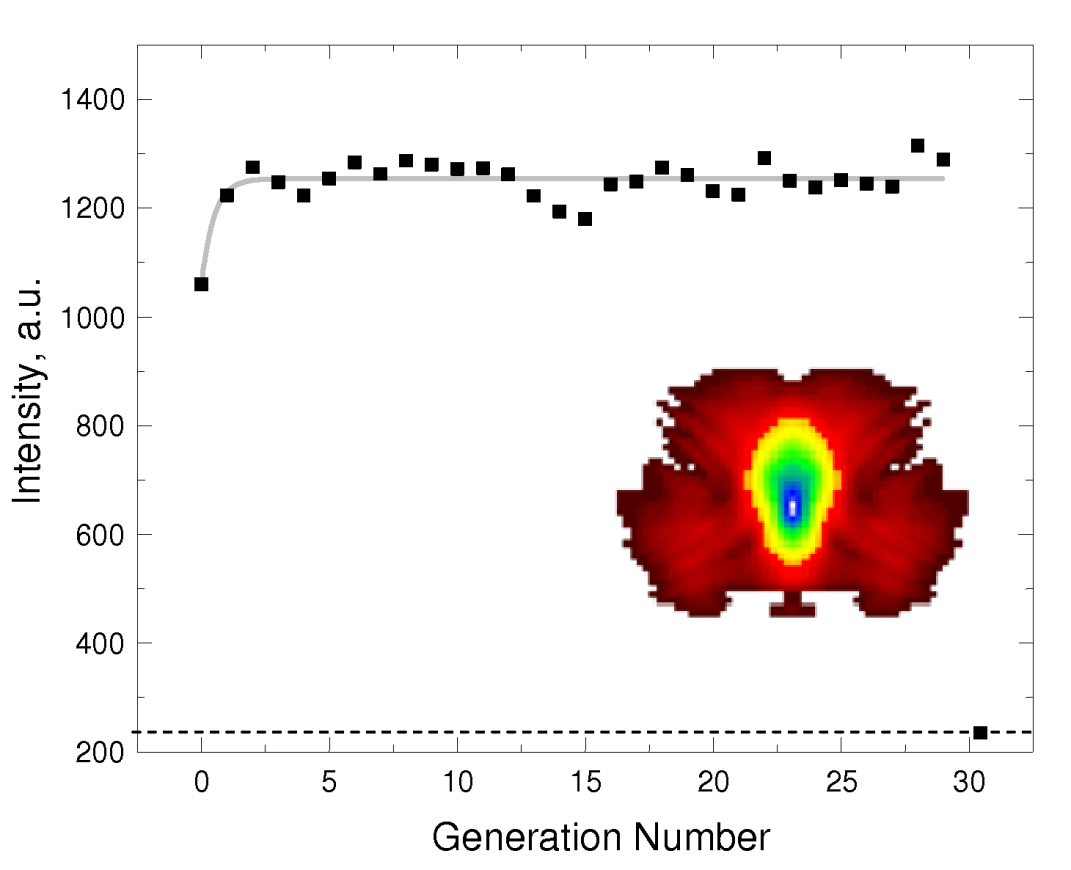


**SI.2.2** The intensity of anthracene fragment ion C_3_H_3_ as a function of optimization generations. The fitting solid line is only to guide the eye. The enhancement is related to the post-ionized intensity by using the unshaped pulse as shown as a dashed line. The inset is the optimal FROG trace, providing a color scale of the intensity as a function of the time delay and optical frequency within the pulse.


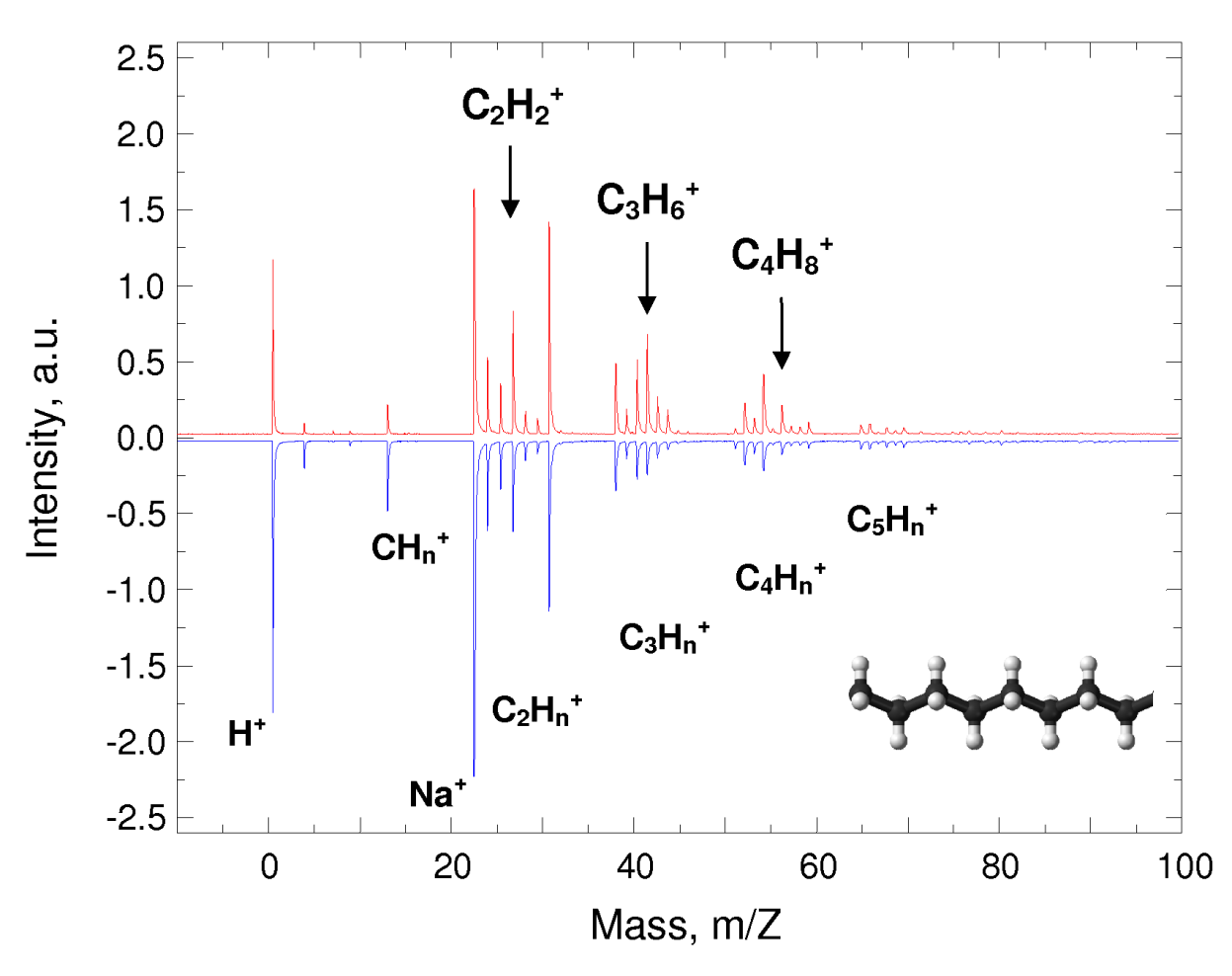


**SI.3.1** Polyethylene mass spectrum post-ionized with unshaped pulse (lower part in blue), the molecular structure as the inset, and adaptively controlled mass spectrum with focus on the C_2_H_2_ fragment with optimized shaped pulse (upper part in red).


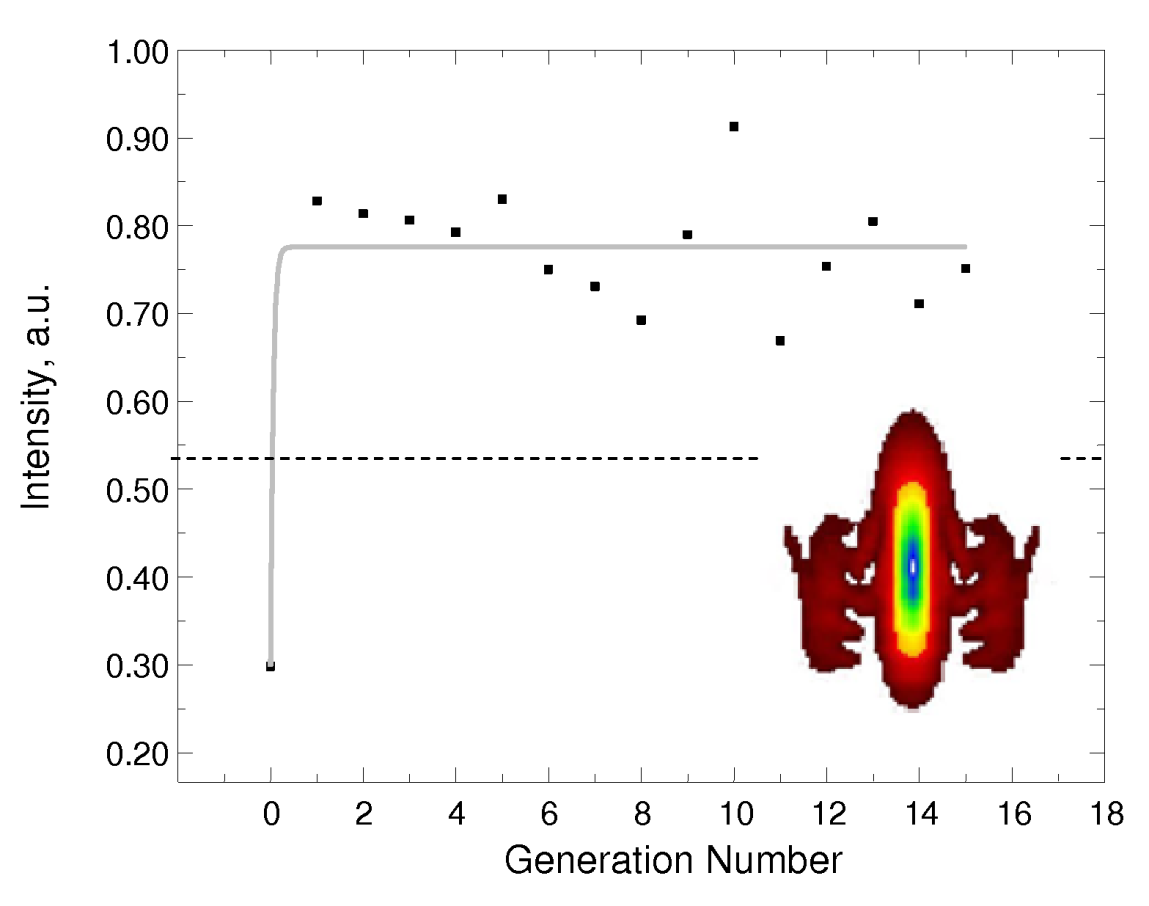


**SI.3.2** The intensity of polyethylene fragment ion C_2_H_2_ as a function of optimization generations. The fitting solid line is only to guide the eye. The enhancement is related to the post-ionized intensity by using the unshaped pulse as shown as a dashed line. The inset is the optimal FROG trace, providing a color scale of the intensity as a function of the time delay and optical frequency within the pulse.


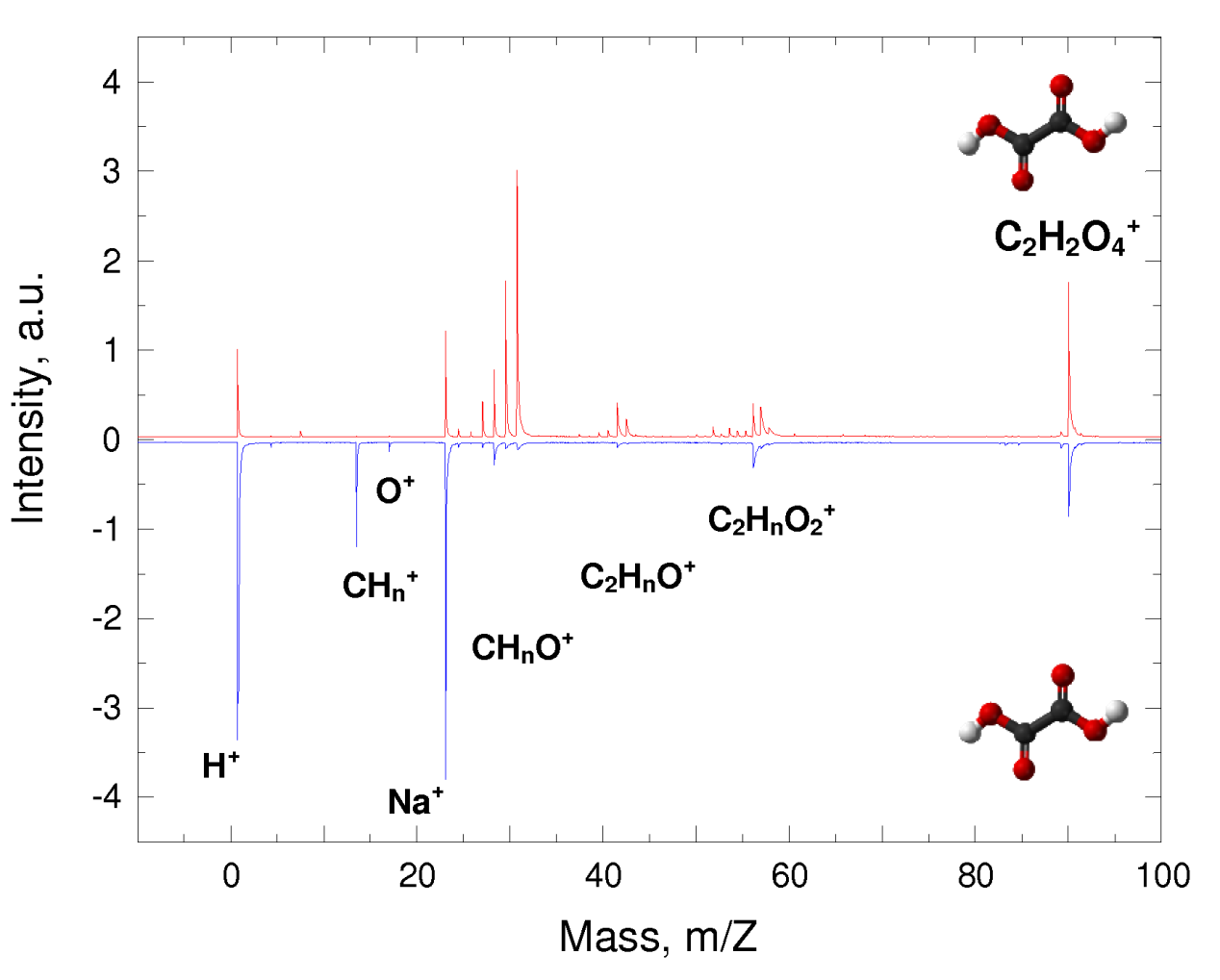


**SI.4.1** Oxalic acid mass spectrum post-ionized with unshaped pulse (lower part in blue), the molecular structure as the inset, and adaptively controlled mass spectrum with focus on the C_2_H_2_O_4_ intact molecular parent ion with optimized shaped pulse (upper part in red), the tentative structure of the molecular parent ion as the inset.


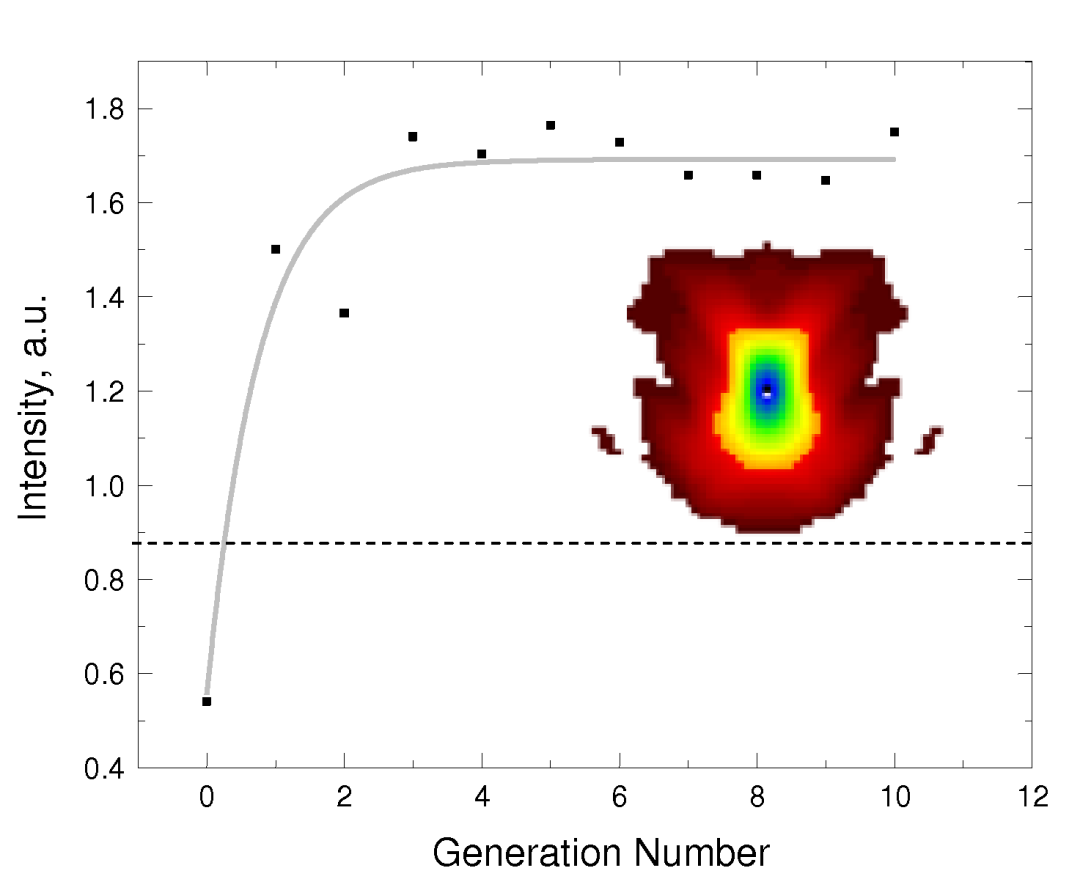


**SI.4.2** The intensity of oxalic acid intact molecular parent ion C_2_H_2_O_4_ as a function of optimization generations. The fitting solid line is only to guide the eye. The enhancement is related to the post-ionized intensity by using the unshaped pulse as shown as a dashed line. The inset is the optimal FROG trace, providing a color scale of the intensity as a function of the time delay and optical frequency within the pulse.
